# The Role of Ultrasound and Shear-Wave Elastography in Evaluation of Cervical Lymph Nodes

**DOI:** 10.1155/2019/4318251

**Published:** 2019-04-30

**Authors:** Jan Heřman, Zuzana Sedláčková, Tomáš Fürst, Jaromír Vachutka, Richard Salzman, Jaroslav Vomáčka, Miroslav Heřman

**Affiliations:** ^1^Department of Otorhinolaryngology, Faculty of Medicine and Dentistry, Palacký University Olomouc and University Hospital Olomouc, I. P. Pavlova 6, Olomouc, Czech Republic; ^2^Department of Radiology, Faculty of Medicine and Dentistry, Palacký University Olomouc and University Hospital Olomouc, I. P. Pavlova 6, Olomouc, Czech Republic; ^3^Department of Medical Biophysics, Faculty of Medicine and Dentistry, Palacký University Olomouc, I. P. Pavlova 6, Olomouc, Czech Republic

## Abstract

**Aim:**

To evaluate the prognostic value of ultrasound and shear-wave elastography (SWE) in diagnosing malignant cervical lymph nodes.

**Methods:**

A total of 99 patients with enlarged lymph nodes (99 lymph nodes presenting as a neck mass) were examined clinically with conventional ultrasound including Doppler examination and shear-wave elastography. The results of the examinations were compared with the final diagnosis.

**Results:**

There were 43 benign and 56 malignant lymph nodes in our cohort. Age and sex were significant predictors of malignancy. The standard ultrasound parameters—node size, long/short axis ratio, hilum, vascularization, and the presence of microcalcifications—were also statistically significant. Lymph node volume combined with age showed the best predictive power. The maximum stiffness found on SWE was also a significant predictor of malignancy. The combination of epidemiologic, classic ultrasound, and elastographic parameters yielded the highest sensitivity and specificity in the prediction of malignancy; however, the additional impact of elastographic parameters was low.

**Conclusion:**

A combination of epidemiologic and classic ultrasound parameters can discriminate between malignant and benign lymph nodes with satisfactory sensitivity and specificity. Examining the stiffness of lymph nodes by means of SWE does not add much new predictive power.

## 1. Introduction

Ultrasound (US) is frequently used as the first imaging modality in diagnosing neck masses because of its availability, safety, and relatively low cost. It may be helpful in distinguishing benign from malignant lymphadenopathy [1], but its performance is suboptimal in many cases and other imaging modalities are recommended by guidelines in specific situations: contrast-enhanced CT or MRI in evaluation of a neck mass suspicious for malignancy [2]; CT or PET-CT in evaluation of lymphoma [3] and even in imaging of the thyroid where US is the unquestioned method of choice; and other modalities such as CT, MRI, or scintigraphy in advanced cancer with suspected metastases or retrosternal growth [4]. Fine needle aspiration biopsy (FNAB) that may be ultrasound-guided uncovers the biological character of the lesion in many cases and may be regarded as golden standard in preoperative diagnostics; however, it is an invasive procedure and may return an inconclusive report.

Some authors question the added value of standard ultrasound in salivary gland tumors over simple clinical examination with palpation by an experienced clinician [5]. The same questions may arise in examining cervical lymph nodes as well.

Elastography is an ultrasonographic method for evaluation of tissue stiffness. Older conventional strain elastography has produced at best semiquantitative estimates of tissue stiffness. The relatively novel method of shear-wave elastography (SWE) offers an advantage of quantitative measurements (tissue stiffness in m/s or in kilopascals, kPa), has lower operator dependence, and shows a relatively narrow range of normal tissue values [6–9]. So far, it has been well established in the staging of liver fibrosis and of breast lesions, possibly also in thyroid gland lesions [10–12] where its use increases the sensitivity and specificity of US examination.

Our literature review revealed eight studies reporting the use of SWE in cervical lymph nodes [1, 13–19]. Only four of them use supersonic SWE with the results in kPa, which may be best compared with our study [1, 13, 14, 17]. However, there are significant variations in the designs of these studies, which make direct comparison nearly impossible. Bhatia [13] enrolled 46 patients with 55 lymph nodes, of which 31 were malignant (4 lymphomas), and, by using the mean elasticity modulus, obtained the best result at a cut-off value of 30.2 kPa, where the sensitivity and specificity were 41.9% and 100%, respectively. Choi [14] studied 67 lymph nodes from 15 patients, with 34 of them being malignant (no lymphomas), and, by using the maximum elasticity modulus with a cut-off value of 19.4 kPa, obtained a sensitivity of 91% and a specificity of 97%. Jung [17] evaluated 84 lymph nodes from 66 patients, with 51 of them being malignant (all papillary carcinoma metastases) and found variable but generally good sensitivity and specificity based on the cut-off value and the stiffness parameter chosen (minimum, mean, and maximum values and elasticity index). Desmots [1] investigated 62 lymph nodes from 56 patients, 30 of which were malignant (including 2 lymphomas). Using the maximum elasticity modulus, he obtained good results especially in the group of subcentimeter malignant lymph nodes.

A rather nice illustration of the confusion in SWE results is the notation of the elasticity modulus: Bhatia uses QboxMax, Choi uses maxSM, Jung uses Emax, and Desmots *μ*_max_, with all of them describing the very same quality—the maximum shear elasticity modulus inside the region of interest.

We have chosen to use the notation maxSM, as it is shorter than QboxMax, is easier to write than *μ*_max_, and, unlike Emax, prevents confusion. A higher Emax implies higher elasticity. In fact, its higher values indicate higher stiffness.

Having a larger cohort of patients and lymph nodes and having included a greater variety of malignant diagnoses, the aim of this study was to investigate the sensitivity and specificity of various prognostic factors in the prediction of the biological behavior of cervical lymph nodes. The prognostic factors evaluated included demographic, standard US, and SWE parameters.

## 2. Materials and Methods

This prospective observational study was approved by the Ethics Committee of the Palacký University and University Hospital Olomouc under reference number 153/13 on December 16, 2013.

We included 99 patients with a neck lump referred for US examination, had it indicated the mass to be a lymph node. A single lymph node only (the most suspicious one on standard US) was identified in each patient and its location was marked on the skin or described precisely if biopsy of the lymph node had been planned. Benign-appearing lymph nodes were not biopsied at all and were followed up for two years. These were considered definitively benign in case of disappearing or showing no progression during follow-up.

The patients mostly underwent surgical removal of the lymph node on the next day after US examination. In cases with a suspected squamous cell metastasis to the neck, the procedure started with a panendoscopy as is a routine practice in cases of carcinomas of unknown primary. If a primary was identified, then the procedure was abandoned. And only after a proper consent was signed by the patient, a primary tumor resection and neck dissection were performed. If a primary was not found, the neck dissection followed after panendoscopy. If a metastasis of well differentiated thyroid carcinoma was suspected, then a total thyroidectomy and neck dissection were performed as a single stage procedure, as is recommended [20]. If preoperative evaluations, i.e., imaging and FNAB, could not direct us towards any diagnosis, lymph node biopsy was done. If a peroperative histology revealed a surprising finding of squamous cell or thyroid carcinoma metastasis, then the procedure was abandoned and completed only after proper discussion with the patient. If a peroperative histology came back as any other pathology (including lymphoma), then a simple lymph node removal was performed.

The FNAB result was taken into consideration when planning or not planning diagnostic lymph node removal. However, the FNAB results were not included in this study as it was firstly not the aim of the study and secondly the SWE result was compared with a definitive histology of the specimen, which was obviously considered superior to FNAB.

The following standard demographic data were obtained from each patient: age, sex, and body mass index (BMI). The cohort comprised 45 women and 54 men, aged 53.4 ± 16.3 years (mean ± standard deviation), range 12–82.

All patients were examined in the supine position by an experienced radiologist performing elastography routinely using the Aixplorer US system (SuperSonic Imagine, Aix-en-Provence, France) with a 4–15 MHz compact linear array transducer. The examination consisted of a conventional US, Doppler US, and SWE with quantitative assessment (Super Sonic Imaging, tissue stiffness measured in kPa). The recorded conventional US features of the lesions included the size in three mutually perpendicular dimensions, margin quality (clearly delineated or blurred), shape (lobular or not), visible hilum (yes/no), presence of microcalcifications (yes/no) and cystic areas (yes/no), laterality (bilateral/unilateral), distal acoustic enhancement (yes/no), or acoustic shadow (yes/no). The number of supplying vessels in the lymph node was also assessed using Doppler US, and the finding was classified as follows: absent/only peripheral vascularization/1–2 vessels/3+ vessels.

**Figure 1:**
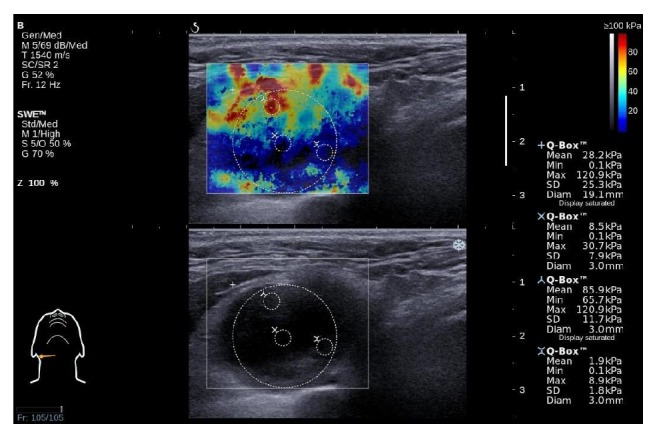
Shear-wave elastography assessment with four regions of interest marked with circles (ROI) inside the lesion. The largest circle denotes the largest ROI, the top center small circle denotes the maximum stiffness ROI, and the bottom right circle denotes the minimum stiffness ROI.

A US device with an SWE module returns the mean, minimum, maximum, and standard deviation (SD) values of the stiffness of a selected region of interest (ROI). For SWE assessment, four circular ROIs were identified. The first ROI was drawn with the largest possible diameter not extending beyond the lymph node margins. A preset circle size was used for the three remaining ROIs. The second ROI was placed in the very center of the tumor, the third one in the area with the highest stiffness, and the fourth one in the area with the lowest stiffness (Figure 1). All the images were stored digitally.

Firstly, demographic data only were used to build a predictive model discriminating benign from malignant lymph nodes. Next, conventional US parameters were assessed, and those shown to be statistically significant in determining the nature of the lymph node were combined with the demographic data to create a refined predictive model discriminating benign from malignant lymph nodes. The predictive powers of various SWE parameters were analyzed. Finally, a model based on demographic parameters and conventional US and SWE predictors was created. The models were built stepwise. The strength of all individual predictors was evaluated by means of univariate analysis (chi-square test or Fisher's exact factorial test in contingency tables). Following that, a multivariate logistic regression model was built. Its sensitivity and specificity were computed for different cut-off levels, and a receiver operating characteristic (ROC) curve was plotted. All the tests were performed using STATISTICA, version 10.0, Statsoft Inc., Tulsa, CA, and MatLab R2013b, The MathWorks Inc., Natick, MA. The level of significance was set at 0.05.

## 3. Results

### 3.1. Cohort Characteristics

**Figure 2:**
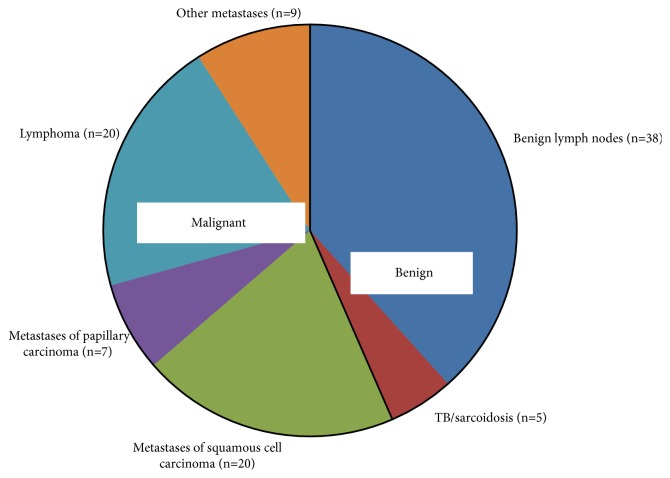
Summary of the diagnoses distribution.

A total of 43 benign and 56 malignant lymph nodes were included in the study; the distribution of the diagnoses is summarized in Figure 2.

Benign lymph nodes included reactive lymphadenitis as well as tuberculous and sarcoid lymph nodes; some features of these groups were also assessed separately. The remaining lymph node diagnoses were regarded as malignant and divided into the following subgroups: squamous cell carcinoma metastases; thyroid carcinoma metastases; lymphoma (which included various types of lymphoma as well as two cases of chronic lymphocytic leukemia); other metastases that included one salivary duct carcinoma and one adenocarcinoma of parotid gland metastases; two adenocarcinomas of prostate gland metastases; adenocarcinoma of esophageal metastasis; carcinoma of gall bladder metastasis; and two metastases of malignant melanoma.

The result of histological examination was available in 84 lymph nodes; 15 benign-appearing lymph nodes were not biopsied. These cases were concluded to be benign lymph nodes after at least two years of follow-up with no progression of lymphadenopathy.

### 3.2. Demographic Parameters

The proportion of benign lymph nodes was higher in women. By contrast, squamous cell carcinoma and distant metastases were more prevalent among men. Sex was a significant predictor of malignancy in our cohort (p = 0.0048). Age was a strong and significant predictor of malignancy as well (p < 0.0001), with a median age of malignant cases of 62.0 years compared to 43.5 years in the case of benign findings. No significant differences in BMI, weight, and height were found between the benign and malignant groups.

### 3.3. Conventional Ultrasound Parameters

Most of the conventional US parameters were good predictors of malignancy in our study: the long-to-short axis ratio was significantly higher in benign cases (p = 0.0007), with a median of 1.91 in benign lymph nodes and 1.53 in malignant ones. The lymph node shape can be approximated by a rotational ellipsoid and its volume estimated as

(1) $$\begin{array}{c} V=\frac{1}{6}\pi \;a\;b\;c \end{array}$$

where* a, b, *and* c* denote the axes of the lymph node. The volume of the lymph node was an excellent predictor of malignancy (p < 0.0001), and malignant findings tended to be several times larger (median volume over 5 cm^3^) than benign ones (median volume 0.85 cm^3^). The absence of a visible hilum predicted malignancy with a p < 0.0001. Excessive vascularization (more than one vessel) predicted malignancy with a p < 0.0001. Microcalcifications were present only in six malignant lymph nodes (five metastases of thyroid carcinoma and one distant metastasis). Thus, the presence of microcalcification was a highly specific but not very sensitive predictor of malignancy.

### 3.4. Elastographic Parameters

**Figure 3:**
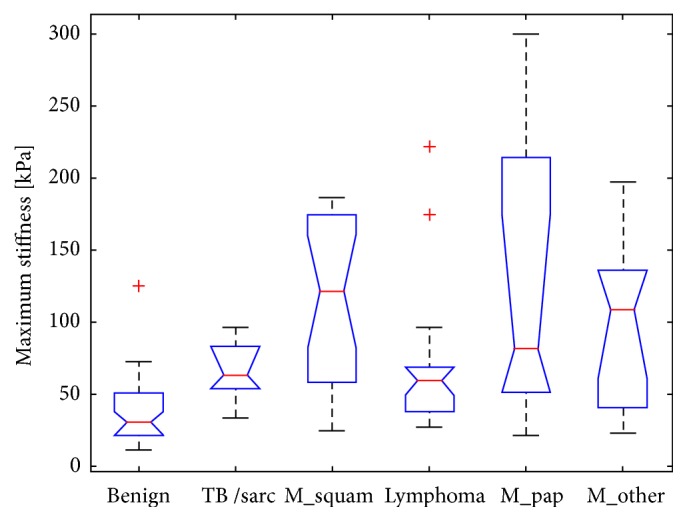
Maximum stiffness is significantly different among the various diagnoses. Malignant findings tend to exhibit higher maximum stiffness with a notable exception of lymphoma (the x-axis: benign lymph nodes; tuberculosis or sarcoidosis; metastases of squamous cell carcinoma; lymphoma; metastases of papillary carcinoma of the thyroid; other metastases).

Elastographic parameters (minimum, mean, and maximum stiffness values) were shown to be significant predictors of malignancy. The maximum stiffness exhibited the most significant difference among the various diagnoses (p < 0.0001; see Figure 3). It is noteworthy that lymphomas disrupted the pattern of malignant findings being generally stiffer than benign ones. The coefficient of stiffness variability (the ratio of the maximum to the minimum stiffness values) [20] also showed significant differences among the diagnoses; however, its discriminatory power was comparable to the maximum stiffness alone.

### 3.5. Predictive Score

**Figure 4:**
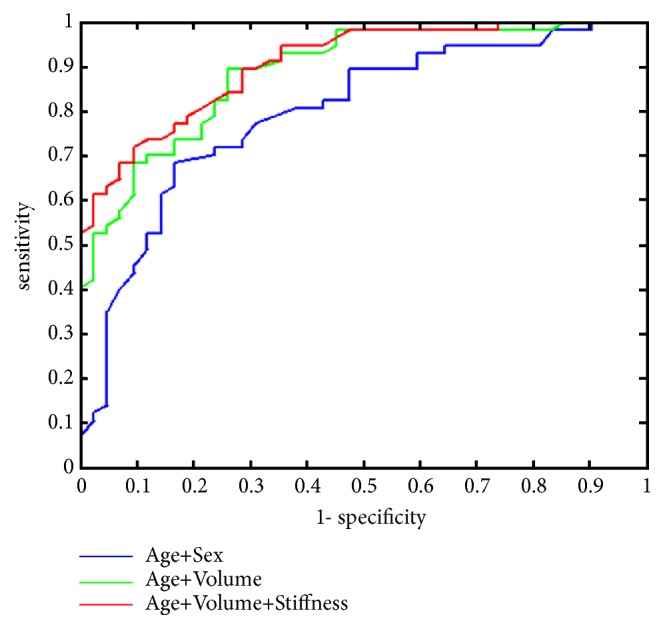
ROC curves for the three predictive models. The blue line predicts malignancy using model (2) which contains age and sex only. The green line shows the ROC characteristics for model (3) which uses age and lymph node volume. Model (4) uses age, lymph node volume, and maximum stiffness. Its ROC curve is shown in red.

We constructed a predictive score based on epidemiologic data, their combination with standard US criteria, and, lastly, their combination with these criteria and elastography. Our aim was to determine whether the finding would be malignant or benign without further distinguishing among the various diagnoses. The purpose of the predictive score was to find how much prognostic value was added by SWE. To start with, we noticed that by using only epidemiologic parameters, a moderately successful predictor could be built. Denoting* Y* the probability of a malignant finding, a suitable epidemiologic logistic regression model, took the form

(2) $$\begin{array}{c} logit\left(\;Y\;\right)=a_{1}+a_{2}*age+a_{3}*female \end{array}$$

where* age *was measured in years and* female *took the value of 1 if the subject was female, and 0 otherwise. In this model, all three parameters were significant (at the level of 0.05). Being female decreased the risk of a malignant finding approximately three times (OR = 0.36, p = 0.03). This effect of sex was approximately matched by the effect of 15 years of age; i.e., a person 15 years older had an approximately three times higher risk of a malignant finding (OR = 2.65 for a 15-year age difference, p = 0.001). The ROC curve for this simple epidemiologic model is shown in blue in Figure 4. It should be noted that even this simple model using no imaging methods at all yields better predictions than estimating malignancy only by its proportions in historical data.

In the second iteration, we used all the classic US parameters combined with demographic parameters, without utilizing the SWE modality. In this case, the trade-off between complexity and performance yields the model

(3) $$\begin{array}{c} logit\left(\;Y\;\right)=a_{1}+a_{2}*age+a_{3}*volume, \end{array}$$

where volume was computed by means of formula (1). The effect of age was approximately retained in this model (OR = 3.1 for a 15-year age difference, p = 0.002). The risk of malignancy increased with an increasing volume of the tumor, with the risk having increased approximately three times for a 10 mm^3^ increase of the volume (OR = 2.7 for a 10 mm^3^ increase of the volume, p = 0.003). There was a high correlation among the classic US predictors; therefore, the final model included only the volume which seemed to carry the strongest information. Figure 4 reveals that the performance of this model is clearly better than that of model (2), although it contains the same number of parameters. Thus, classic US parameters do provide significant prognostic information.

Adding the elastographic parameter maxSM (i.e., the maximum stiffness in the ROI) to model (3) yielded the following prediction:

(4) logitY=a1+a2∗age+a3∗volume+a4∗maxSMs

The effect and significance of age and volume remained the same as in model (3). The effect of stiffness was at the border of significance (p = 0.05). An increase in stiffness by 100 kPa increased the risk of a malignant finding about fourfold (OR = 4.6 for a 100-kPa increase in stiffness, p = 0.05). Predictor (4) was more complex than predictor (3) because it contained three predictors instead of two, yet its performance was not clearly superior to predictor (3); see the green and red lines in Figure 4. For perfect specificity (i.e., no false-positive prediction, specificity = 1), the sensitivity of model (4) was still 0.53, which is better than model (3). On the other hand, at the more important end of the ROC curve (perfect sensitivity, i.e., no false-negative predictions), models (3) and (4) are comparable.

## 4. Discussion

The main promise of elastography in predicting the biological nature of a lesion has been based on the premise that malignant tumors have higher stiffness than benign ones. This works well in the case of the breast and thyroid gland [11, 12]. However, the situation in cervical lymph nodes and other organs (e.g., the parotid gland; see [21]) appears to be more complicated. This is caused by the very variable histoarchitecture of these lesions, which results in considerable variance in stiffness found both in our study (Figure 3) and in previously published papers [1, 13, 14].

As for the notations of the shear modulus (the outcome of supersonic elastography, in kPa) that have been used in the papers published, we prefer the notation SM, since it is easy to write and prevents confusion [1, 13, 14, 17].

There are profound differences in the reported predictive power of SWE among various studies based on the selected population and the groups of lymph nodes. Lymphomas were not generally stiffer than benign lymph nodes in our study, unlike other malignant findings. Thus, one may expect that the performance of SWE predictors in various cohorts will depend on the proportion of lymphoma in the cohort studied.

The comparisons between healthy lymph nodes and those suspicious for squamous cell carcinoma or thyroid cancer metastases yield much better sensitivity and specificity than the comparison of inflammatory lymph nodes and lymphoma, even though both of these settings may be regarded as a benign-versus-malignant discrimination [1, 7, 17] (Figure 3).

Combined information about age and sex had a relatively good accuracy in predicting malignancy in our study (Figure 4). In male patients over 40 years of age with a neck mass, at most one antibiotic should be prescribed before a thorough diagnostic process, and, until excluded clearly, malignancy should always be suspected as its proportion is high in this group [22]. However, since lymphomas and thyroid carcinomas may be present in pediatric age as well, adequate diagnostic evaluation is important at any age.

Conventional US may bring an advantage in preoperative prediction of histological outcome over basic demographic data: age and sex (Figure 4). Ultrasound elastography improves accuracy somewhat further, but as the enhancement is minimal, factors such as time of the examination, need for special training, and cost of the device should be considered.

## 5. Conclusion

The sensitivity and specificity of standard ultrasound parameters in discriminating benign from malignant cervical lymph nodes were shown to be somewhat better than a qualified estimate based on epidemiologic data. In our study, elastography (maximum stiffness) improved the performance of standard ultrasound marginally only. The type of malignancy and the number of lesions evaluated influence the results of elastography studies of cervical lymph nodes.

## Data Availability

The data used to support the findings of this study are available from the corresponding author upon request.
